# Proteolytic modulation of tumor microenvironment signals during cancer progression

**DOI:** 10.3389/fonc.2022.935231

**Published:** 2022-09-05

**Authors:** Yoshifumi Itoh

**Affiliations:** Kennedy Institute of Rheumatology, University of Oxford, Oxford, United Kingdom

**Keywords:** TME, proteinases, ECM, invasion, matrikine, Soluble factors, membrane protein shedding

## Abstract

Under normal conditions, the cellular microenvironment is optimized for the proper functioning of the tissues and organs. Cells recognize and communicate with the surrounding cells and extracellular matrix to maintain homeostasis. When cancer arises, the cellular microenvironment is modified to optimize its malignant growth, evading the host immune system and finding ways to invade and metastasize to other organs. One means is a proteolytic modification of the microenvironment and the signaling molecules. It is now well accepted that cancer progression relies on not only the performance of cancer cells but also the surrounding microenvironment. This mini-review discusses the current understanding of the proteolytic modification of the microenvironment signals during cancer progression.

## 1 Introduction

It is now well accepted that surrounding microenvironment is a determinant of the cancer cell progression (1). Cancer cells modify a normal tissue microenvironment and turn into a tumor microenvironment (TME) that helps cancer cells to grow, invade, and metastasize (1). A major component of the TME is the extracellular matrix (ECM) (2, 3). Cancer cells recognize ECM components through ECM receptors and modify them by depositing or degrading the ECM (3). Cells within the TME, such as cancer-associated fibroblasts (CAFs) and tumor-associated macrophages (TAMs), also contribute to this process. In addition to being a glue function to connect the cells and a bordering function to separate tissues and organs, the ECM also acts as signaling molecules, a pool for cytokines and growth factors, and a scaffolding for migrating cells (3). Thus, the modification of the pericellular ECM would impact cancer progression significantly.

**Figure 1:**
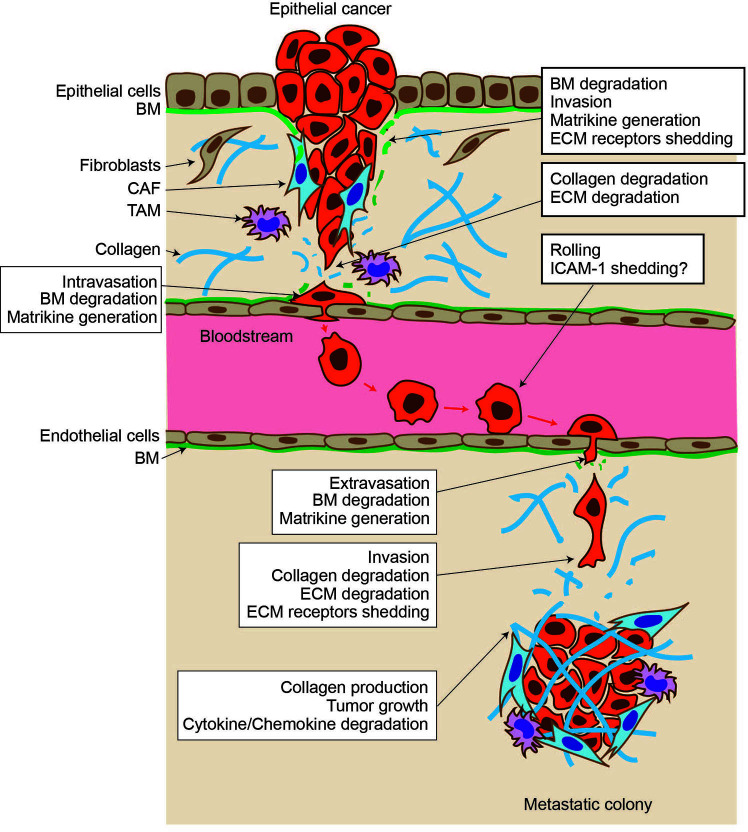
Proteolytic events during cancer progression. Epithelial cancer arises within the epithelial layer. They break through the basement membrane (BM) and invade stromal tissue. Upon BM degradation, matrikines are generated by proteolytic action, and cell surface extracellular matrix (ECM) receptor shedding promotes cancer cell motility. Stromal fibroblasts are activated and become cancer-associated fibroblasts and help cancer cells further invade. Tumor-associated macrophages (TAMs) help to evade the immune system. Cells degrade stromal the ECM further, including type I collagen, and intravasate into the vessel. Cancer cells traverse other organs through the bloodstream, interacting and rolling on the endothelial cell layer. Intercellular adhesion molecule–1 may be expressed in cancer cells, and its shedding allows cancer cells to migrate through the endothelial cell layer and extravasate. Cancer cells invade stromal tissue, form a metastatic colony, create a tumore microenvironment, and cause tissue malfunction.

Invasion and metastasis are the most life-threatening feature of malignant cancers. Transformed epithelial cells gain the ability to proliferate, downregulate cell–cell adhesion, and degrade the basement membrane (BM) and stromal tissue under it. This invasion process disturbs tissue architecture and causes the loss of tissue function. Once cancer cells reach either blood vessels or lymphatic vessels, they intravasate and traverse to distant organs. During this process, many cancer cells are attacked by immune cells. Still, some cancer cells evade this immune surveillance and reach the organ where they can extravasate and create metastatic colonies. Once they extravasate, they grow, invade tissue, and cause tissue malfunction (**Figure 1**).

One of the means for cancer cells to modify their microenvironment signals is by using proteolytic enzymes. These proteinases degrade the ECM for growth, invasion, metastasis, and causing tissue damage (4). Proteinases can also generate bioactive fragments from the ECM by limited processing. It also degrades or processes soluble factors and modifies the signaling events in cancer cells and other cells within the TME. Proteinases also shed transmembrane (TM) cell surface receptors for cytokines and growth factors and adhesion molecules for cell–cell and cell–ECM attachments and adhesion. Proteolytic events are non-reversible reactions and are thus effective in modulating the function of the molecules. This mini-review discusses the current understanding of proteolytic modification of microenvironment interaction in cancer.

## 2 Proteolytic enzymes involved in microenvironment modulation

### 2.1 Matrix metalloproteinase

**Figure 2:**
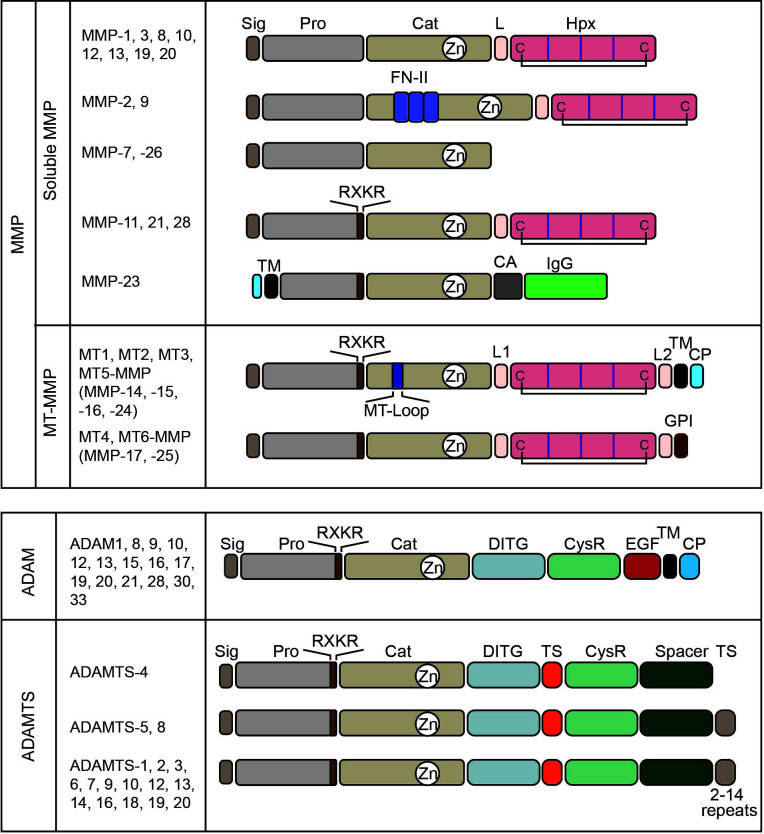
Domain structure of metalloproteinases. Matrix metalloproteinases (MMPs) can be divided into two major groups: soluble MMPs and membrane-type MMPs. According to their structures, they can be classified into six soluble MMPs and two subgroups in membrane-type MMPs. MMP-11, 21, 28, 23, and MT-MMPs have a basic motif of RXKR that is recognized and cleaved by proprotein convertases to activate the enzymes by removing their pro-domain. Sig, signal peptide; Pro, pro-domain; Cat, catalytic domain; L, linker or hinge region; Hpx, hemopexin domain; C, cysteine; FN-II, fibronectin type-II repeats; TM, transmembrane domain; CA, cysteine array; IgG, IgG-like domain; L1, linker1 or hinge region; L2, linker 2 or stalk region; MT-Loop, eight amino acids insertion unique to TM-type MT-MMPs; and CP, cytoplasmic domain. ADAM enzymes have a conserved domain structure. DITG, a disintegrin-like domain; CysR, a cysteine-rich domain; EGF, an EGF-like domain. ADAMTS enzymes also have a conserved domain structure and differ in the number of thrombospondin motifs (TS) at their C-terminus. ADAMTS-4 is the smallest, without a C-terminal TS, and ADAMTS-5 and 8 have two. Other members have 2–14 repeats. Spacer, spacer domain. Both ADAM and ADAMTS enzymes have an RXKR motif at the C-terminus of their propeptide for activation by proprotein convertases.

Matrix metalloproteinases (MMPs) belong to the metzincin clan of metalloendopeptidase, and the major substrates of MMPs are ECM components (5). They harbor zinc in their catalytic site for the hydrolysis of peptide bonds. There are 23 MMPs in humans, and they can be divided into two groups according to their membrane-bound nature: soluble MMPs and membrane-type MMPs (**Figure 2**) (6). Within soluble MMPs, they can be further classified according to domain structure. Classical MMPs contain a pre-/propeptide, a catalytic domain, a hinge, and a hemopexin domain. This group contains interstitial collagenase (MMP-1), stromelysin 1 (MMP-3), neutrophil collagenase/collagenase 2 (MMP-8), stromelysin 2 (MMP-10), macrophage elastase (MMP-12), collagenase 3 (MMP-13), MMP-19, enamelysin (MMP-20), and MMP-27. Gelatinase MMPs have three repeats of the type II fibronectin domain in the catalytic domain, allowing them to bind collagen and gelatin. This group has two gelatinases, MMP-2 (gelatinase A) and MMP-9 (gelatinase B). The third group is minimal as its domain structure consists only of a pre-/propeptide and a catalytic domain. Matrilysin 1 (MMP-7) and matrilysin 2 (MMP-26) belong to this group. The fourth group has classical MMPs’ domain structure, but it has a basic amino acid motif of R-X-K/R-R that is recognized and cleaved by proprotein convertases (PCs) such as furin at the C-terminus of the propeptide, allowing them to be activated during the secretory process by PCs. This group includes stromelysin 3 (MMP-11), MMP-21, and epilysin (MMP-28). MMP-23 is a unique member of MMP that is a type II TM enzyme. The TM domain is located at the N-terminus of the propeptide, and the basic motif of R-R-R-R for activation by furin is inserted at the C-terminus of the propeptide. Thus, MMP-23 becomes a soluble enzyme upon activation. Membrane-type MMPs (MT-MMPs) have two subgroups. One is type I TM-types, including MT1-, MT2-, MT3-, and MT5-MMPs, and the other glycosylphosphatidylinositol (GPI)-anchored types, including MT4- and MT6-MMPs. As they have a membrane-anchored part at the C-terminus of the enzyme molecule, MT1-MMPs are expressed on the cell surface. All MT-MMPs have the basic motif of R-X-K/R-R motif at the C-terminus of propeptide for activation by PCs. TM-type MT-MMPs have a characteristic eight amino acid insertion in the catalytic domain called MT-Loop or IS-2. GPI-type MT-MMPs do not have this insertion.

### 2.2 A disintegrin and a metalloproteinase

ADAM belongs to the metzincin clan of metalloendopeptidase-like MMPs. There are 12 ADAMs that are catalytically active and eight ADAMs that are catalytically inactive (**Figure 2**) (7). ADAMs are type-I TM proteinases and share conserved domain structures within the family. They consist of a signal peptide, pro-domain, catalytic domain, disintegrin domain, cysteine-rich module, EGF-like domain, TM domain, and cytoplasmic domain. ADAMs also have a basic R-X-K/R-R motif at the C-terminus of propeptide for activation by PCs at trans-Golgi. Thus, ADAMs are displayed on the cell surface as an active form. ADAMs are generally considered membrane protein sheddases, cleaving a range of cell surface proteins, including cytokines, growth factors, receptors for cytokines and growth factors, and cell adhesion molecules (8). Among 12 catalytically active ADAMs, ADAM10 and ADAM17 are the most characterized dominant membrane sheddases.

### 2.3 A disintegrin and metalloprotease with thrombospondin type 1 motif

ADAMTS enzymes have conserved domain structures, consisting of a signal peptide, pro-domain, catalytic domain, disintegrin domain, cysteine-rich module, spacer domain, and additional repeats of thrombospondin regions for some enzymes (**Figure 2**) (9). ADAMTS enzymes also have a basic motif of R-X-K/R-R motif at the C-terminus of the pro-domain and are activated by PCs during secretion. Unlike ADAMs, ADAMTSs are soluble enzymes whose primary function is ECM degradation (9).

### 2.4 Plasmin system

**Figure 3:**
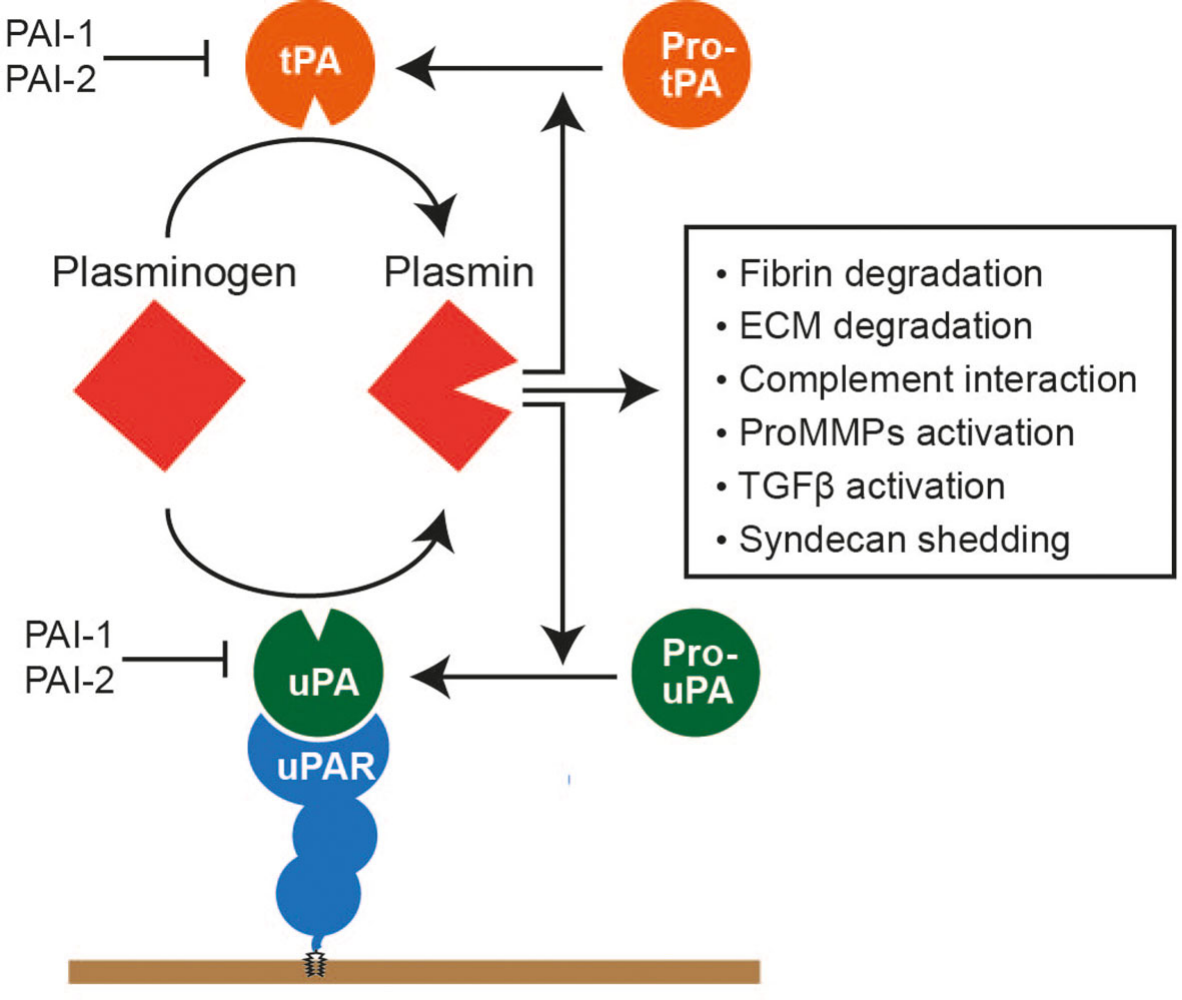
Plasmin system. Plasminogen is a precursor form of plasmin, mainly produced in the liver and present in the plasma at approximately 150–200 μg/ml. Plasminogen is activated by either a tissue plasminogen activator (tPA) or urokinase plasminogen activator (uPA). uPA is bound to the glycosylphosphatidylinositol-anchored uPA receptor and activates plasminogen on the cell surface. tPA and uPA are also produced as precursor forms, and activated plasmin can activate these activators. Activated plasmin can degrade cross-linked fibrin and various ECM components, interact with the complement system to facilitate complement cascade, activate proMMPs, activate the precursor form of TGFβ, and cause syndecan shedding.

The serine proteinase plasmin is the major proteinase in our body fluid (10). It is mainly produced in the liver and exists in the plasma as its precursor form, plasminogen (or Glu plasminogen with the glutamic acid at the N-terminus), at approximately 0.2 mg/ml. Its major function is fibrin degradation, but it also degrades ECM components, including fibrinogen, laminin, vitronectin, and osteocalcin. It also activates several proMMPs, including proMMP-1, proMMP-3, proMMP-9, and proMMP-13. It cleaves complement components 3 and 5 (C3 and C5), factors V, VIII, and X, and protease-activated receptors (PARs). Thus, plasmin possesses a broad substrate specificity. Plasminogen is activated by two types of plasminogen activators (PA), urokinase PA (uPA) and tissue-PA (tPA), by the cleavage in the activation loop between Arg_561_ and Val_562_. Activated plasmin activates pro-uPA and pro-tPA, providing positive proteolytic feedback (**Figure 3**).

### 2.5 Neutrophil-derived serine proteinases

Neutrophil in the TME produces serine proteinases, including neutrophil elastase (NE), proteinase 3 (Pro3), and cathepsin G (CG) (11, 12). NE, Pro3, and CatG cleave elastin, the telopeptide region of fibrillar collagen types I, II, III; collagen types IV, VI, VIII, IX, X, and XI; and fibronectin, laminin, and aggrecan (11, 12). They can also activate proMMPs and inactivate endogenous proteinase inhibitors such as α2 antiplasmin, α1 antichymotrypsin, and the tissue inhibitors of metalloproteinases (TIMPs) (11, 12). It has also been shown that NE and CatG activate PARs (11, 12).

## 3 Microenvironment modification by proteolytic enzymes

### 3.1 Extracellular matrix degradation for cancer invasion

The ECM holds cancer and other cells together to create a TME. The ECM provides signals to cells through ECM receptors and migration cues. On the other hand, the ECM also acts as a physical barrier to invading cancer cells as it is a solid matrix. For a cancer cell to migrate through the ECM, its pore size needs to be big enough for cells to squeeze the nuclei to migrate through: 7 μm^2^ for cancer cells (13). If the ECM gaps are not sufficiently large, cells will use proteolytic enzymes to enlarge the opening to migrate through. It has been well accepted that one of the major proteinases to degrade the ECM during invasion under the condition is MT1-MMP (4, 13–15). MT1-MMP degrades many ECM components, including fibrillar collagens I, II, III, fibronectin, vitronectin, laminins-1, -2, -4, and -5, fibrin/fibrinogen, perlecan, and aggrecan (4, 14). It activates other MMPs on the cell surface, namely, proMMP-2 and proMMP-13, expanding the proteolytic repertoire (14). ProMMP-2 activation is critical when cancer cells need to degrade the BM since a major component of the BM can be degraded by activated MMP-2 but not by MT1-MMP itself. It was shown that epithelial cancer cells could not invade or grow without stromal-derived proMMP-2 due to the inability to degrade type IV collagen (14, 16). MT1-MMP is also a major collagenase that promotes cancer invasion in stromal tissue. There are five collagenolytic MMPs, including MMP-1, MMP-2, MMP-8, MMP-13, and MT1-MMP. However, MT1-MMP is the only collagenase that promotes cellular invasion within the type I collagen matrix (17).

MT1-MMP is regulated by different post-translational mechanisms to promote cellular invasion effectively. One is homodimer formation on the cell surface through the hemopexin (Hpx) domain and TM domain (18, 19). The homodimer state is considered an active state of the enzyme, as both the proMMP-2 activation and fibrillar collagen degradation activities of MT1-MMP on the cell surface require the enzyme to be in the dimer state (18, 20). In invading cells, the dimerization of MT1-MMP constitutively occurs at the leading edge (21). The dimerization was regulated by the coordination of the actin cytoskeleton, and Rac1 and Cdc42 activation was shown to enhance MT1-MMP dimerization (21).

The second regulation is localization at the “leading edge” membrane structures, such as lamellipodia and invadopodia. Preventing MT1-MMP localization to the leading edge would disable MT1-MMP-dependent cancer invasion as the coordination of MT1-MMP activity and migrating machinery would be lost (15). It was shown that MT1-MMP localization at the lamellipodia is mediated by interacting with a hyaluronan receptor CD44 through the Hpx domain (22). Since CD44 is associated with the actin cytoskeleton through ERM proteins at the cytoplasmic domain, MT1-MMP is also associated with F-actin indirectly.

Invadopodia is another leading-edge membrane structure, and it was initially characterized as “a vertical membrane protrusion extends towards ECM that contains proteinases to degrade ECM” (23). The key components of the invadopodium include a scaffold protein, tyrosine kinase substrate with 5 SH3 domains (Tks5), the actin-regulating molecule cortactin, neural Woskott–Aldrich syndrome protein N-WASP, and cofilin (23). Aside from these molecules, MT1-MMP is another component that provides invasive function to the membrane structure (23). It has been extensively documented that breast cancer cells extend the invadopodia structure and degrade the matrix creating punch hole degradation spots (24–29). However, as the invasion process progress, a single protrusion seems to expand to a larger cell cortex along the matrix to degrade further and push the ECM to expand the gaps of the meshwork (27). Thus, invadopodia can no longer be defined as a protruding membrane structure but as a membrane with a molecular composition of Tks5, cortactin, F-actin, and MT1-MMP. It was shown that MT1-MMP localization at the invadopodia requires the cytoplasmic tail (CT) of the MT1-MMP (27, 30), suggesting that the CT-binding molecules play a role in MT1-MMPp localization at invadopodia. However, the crucial adaptor molecule enabling MT1-MMP localization at the invadopodia has not been identified yet.

**Figure 4:**
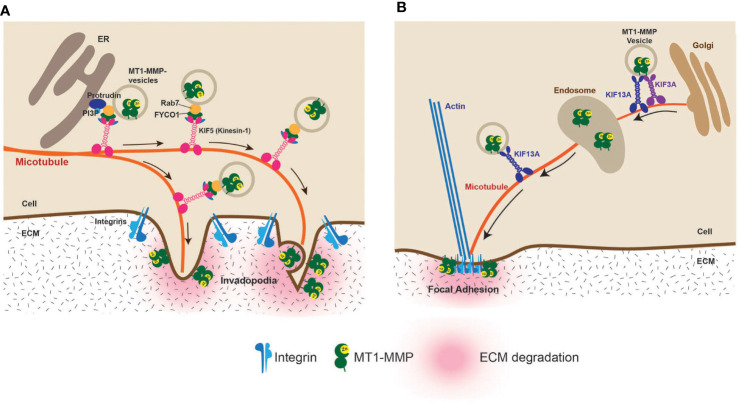
Vesicle transport of MT1-MMP to the invadopodia and the focal adhesion (FA). **(A)** Vesicle transport of MT1-MMP to the invadopodia has been extensively studied. It was shown that the endoplasmic reticulum protein protrudin plays a crucial role. Protrudin makes contact sites with RAB7 and phosphatidylinositol 3-phosphate (PI3P)–positive MT1-MMP-containing vesicles. Protrudin hands over RAB7-binding KIF5 adaptor protein FYCO1, enabling the transport of MT1-MMP-containing vesicles along microtubules toward invadopodia (32). **(B)** It was found that localization at FA is due to the direct transport of MT1-MMP-containing vesicles along the microtubules. KIF3A and KIF13A transport the vesicles between the *trans-Golgi* and the endosome. From the endosome, KIF13A solely transports the vesicles to the plasma membrane. The FA localization of MT1-MMP is essential for the HT1080 cell invasiveness (33).

On the other hand, two kinesin motor proteins, Kinesin-1 (KIF5B) and Kinesin-2 (KIF3A), were shown to be involved in MT1-MMP trafficking to the invadopodia (31). Recently, the picture of the MT1-MMP vesicle trafficking to invadopodia was further clarified by a study reporting that the ER (endoplasmic reticulum) protein, protrudin, is crucially involved in MT1-MMP vesicle transport to the invadopodia (**Figure 4A**) (32). Protrudin makes contact sites with RAB7 and phosphatidylinositol 3-phosphate (PI3P)–positive late endosomes (LEs) containing MT1-MMP. Protrudin hands over RAB7-binding KIF5 adaptor protein FYCO1, enabling the transport of MT1-MMP-containing vesicles along microtubules toward invadopodia at the plasma membrane (**Figure 4A**) (32).

Aside from invadopodia, MT1-MMP has been shown to localize at the focal adhesion (FA) (34–36). FA is where the distance of the plasma membrane and ECM is the closest since integrins directly interact with ECM components at FA. However, unlike invadopodia localization, MT1-MMP localization at the FA is independent of the CT of the MT1-MMP (34). It was shown that an eight-amino-acid loop structure called MT-Loop or IS-2 (^163^PYAYIREG^170^) within the catalytic domain is necessary to localize at the FA (34). Since MT-Loop is positioned on the opposite side of the catalytic site in the catalytic domain, it does not influence the catalytic function. Still, it acts as a molecular interface, allowing MT1-MMP to localize at the FA (34). It was recently discovered that the FA localization of MT1-MMP is carried out by direct intracellular trafficking of MT1-MMP-containing vesicles to the FA by kinesin superfamily motor proteins, KIF13A and KIF3A (33). KIF3A and KIF13A coordinately transport MT1-MMP-containing vesicles from the *trans-Golgi* to endosomes. KIF13A alone then takes over the vesicles and transports them from the endosomes to the plasma membrane (33) (**Figure 4B**). This is a distinct vesicle trafficking pathway from invadopodia as KIF5B is not involved (33). It is possible that KIF3A- and KIF13A-mediated trafficking can be therapeutic targets to prevent invasion in specific cancers that utilize FA-dependent invasion mechanisms.

In contrast to ECM degradation, the stiffening of the tumor ECM, called desmoplasia, is also known to correlate with tumor aggressiveness. In this case, stiffened ECM-driven signals promote the progression of cancer. This aspect is reviewed by Gkretsi and Stylianopoulos (37). It is seemingly contradictory, but cancer invasion only requires local ECM degradation; thus, it makes sense.

### 3.2 Generation of matrikines: Bioactive fragments from extracellular matrix

ECM components are generally large multidomain glycoproteins that interact with each other to create a unique solid structure to support the function of the cells, tissues, and organs (2). These ECM molecules contain hidden sequences or modules that can send signals to the cells through the receptors upon exposure. These epitopes can be revealed either due to partial unfolding of the protein structures or proteolytic processing (38). These ECM fragments are termed “matrikines” (39). This section discusses collagen-derived fragments, elastin-derived fragments, and laminin-derived fragments.

#### 3.2.1 Collagen-derived fragments

Non-fibrillar collagens consist of triple helical regions and non-triple helical regions, and some of these collagens were shown to contain antiangiogenic fragments. The first example is type XVIII collagen. It is a component of the BM and plays a significant role in providing the integrity of the structure of the BM for both endothelial and epithelial cells. It is well known that type XVIII collagen proteolysis generates angiogenesis inhibitor endostatin (38). It is a C-terminal non-collagenous domain 1 (NC1) fragment of collagen XVIII. Endostatin can be generated by many different proteinases, including the lysosome cysteine proteinases of Cathepsin L, Cathepsin B, and Cathepsin K; MMPs including MMP-3, MMP-9, MMP-12, MMP-13, MMP-20; and, to a less extent, by MMP-2 and MT1-MMP (38). An increase in the proteolytic activities of these proteinases in tumor tissue would generate endostatin and delay tumor angiogenesis and thereby tumor growth and metastasis.

The following example is type IV collagen. It is a major BM component and forms a thin sheet-like structure with laminin 5. Type IV collagen is composed of six different α chains (α1–α6) that are encoded by six different genes (COL4A1–COL4A6) (40). The three primary antiangiogenic fragments released from the α1, α2, and α3 chains of type IV collagen are arresten, canstatin, and tumstatin, respectively (40). It was shown that arresten was generated upon p53 activation in cancer cells, and it was due to p53-induced MMP activity (41). It was reported that MMP-2, MMP-3, and MMP-13 could generate tumstatin, but MMP-9 was the most efficient in liberating it from type IV collagen, and MMP-9 null mice showed significantly decreased circulating blood levels of tumstatin (42). While endostatin inhibits both physiological and tumor angiogenesis, tumstatin inhibits only tumor angiogenesis. It is because the tumstatin’s receptor αvβ3 only plays a role in tumor angiogenesis (42). It was also shown that MT2-MMP could cleave the NC1 domain of type IV collagen in the submandibular gland, which promotes branching morphogenesis (43). Since MT2-MMP cleaves off all three chains, it effectively generates arresten, canstatin, and tumstatin (43).

The final example of antiangiogenic fragment generation is type XV collagen. It is classified as a chondroitin sulfate proteoglycan and a member of the multiplexin and non-fibrillar collagen subgroups (44). It is also a member of the FACIT collagen family (fibril-associated collagens with interrupted helices). Upon the cleavage of the C-terminal NC1 domain of type XV collagen on its α1 chain, restin, a 22-kDa antiangiogenic factor similar to endostatin, is produced (45). Restin exhibits antiangiogenic properties *in vivo* in xenograft carcinoma mouse models (45). However, responsible enzymes to generate restin have not been described. Similar other antiangiogenic fragments of ECM molecules include vastatin (type VIII collagen), anastellin (fibronectin), and endopellin (perlecan), but responsible proteinases are not known.

#### 3.2.2 Elastin-derived fragments

Elastin provides elasticity and resilience to tissues, including the lungs, arteries, and skin. It shows a unique protein containing a large amount of four hydrophobic amino acids of Gly, Val, Ala, and Pro. It is heavily cross-linked at Lys residues. Due to its hydrophobicity and extensive cross-linking, elastin is insoluble, highly resistant to proteolytic degradation, and does not undergo substantial turnover in healthy tissue (46–48). However, it was found that elastin can be extensively degraded by proteinases related to inflammation and cancer.

It has been reported that MMP-7, MMP-9, and MMP-12 degraded elastin extensively and also generated fragments possessing pro-inflammatory activities (49). It has also been reported that neutrophil-derived serine proteinases, NE, CatG, and RR3, degrade elastin, and these elastin-derived peptides possess pro-inflammatory activities (50). VG-6 (VGVAPG) and AG-9 (AGVPGLGVG) peptides are especially considered to be active fragments showing various pro-inflammatory and protumorigenic activities (38).

#### 3.2.3 Laminin-derived fragments

Laminin has also been extensively studied for biological fragments. Laminin 5 (Laminin 332) is a major component of the epithelial and endothelial BM aside from type IV collagen, and it is composed of the three laminin chains of α3, β3, and γ2. After secretion and deposition in the ECM, laminin 332 undergoes physiological maturation processes consisting of the proteolytic processing of domains located within the α3 and the γ2 chains by plasmin (51), mammalian tolloid (mTLD) (52), and bone morphogenic protein 1 (BMP-1) (53). These maturation events are essential for laminin 332 integration into the BM, where it plays a vital function in the nucleation and maintenance of anchoring structures. C-terminal globular domains 4 and 5 (LG45) of the α3 chain are proteolytically removed during maturation, but soluble LG45 has biological functions. It was shown that soluble LG45 induced keratinocyte migration and the expression of MMP-1 and MMP-9 (54).

It was shown that a 30-kDa γ2 chain fragment containing an epidermal growth factor (EGF)–like motif (DIII, domain III) was released by the two cleavages by MT1-MMP and MMP-2 (55–57). The released DIII fragment of the γ2 chain then promotes the migration and invasion of cancer cells by engaging to the EGF receptor (56–58). It is considered that this event promotes the growth and initial breach of the BM at primary tumor sites.

### 3.3 Releasing extracellular matrix–bound soluble factors

One of the important biological roles of the ECM is pooling growth factors and cytokines (59, 60). There are growth factors associated with heparan sulfate (HS) and those that interact with matrix proteins (59, 60). They are secreted from the cells and retained in the ECM. Binding to the ECM prevents these soluble factors from binding to their receptors, but upon liberating from the ECM, they become bioavailable to cells to transmit the signals (59, 60). Proteolytic enzymes are responsible for this release.

One of the examples is the vascular endothelial growth factor (VEGF). The VEGF is a potent inducer of angiogenesis, implicated in cancer angiogenesis (61). It binds to HS through the heparin-binding domain with basic motifs encoded by exons 6 and 7 (61). VEGF has several alternative splicing variants, including VGFA_121_, VGFA_165_, VEGF_189_, and VEGF_206_. VEGF_189_ and VEGF_206_ have two heparin-binding sites, and VEGF_165_ has a single heparin-binding domain encoded by exon 7, while VGFA_121_ does not have the heparin-binding domain (61). VEGF_165_ is the most physiologically relevant VEGF isoform, and the heparin-binding domain locates at the C-terminus. While VEGF_189_ and VEGF_206_ exclusively localize on the cell surface or ECM and cannot be detected in the culture medium due to two heparin-binding domains, 50%–70% of VEGF_165_ can be secreted to the medium due to weaker affinity to HS (62). Serine proteinase plasmin was shown to proteolytically release the ECM-bound VEGF species of both VEGF_165_ and VEGF_189_ into a soluble biologically active VEGF (63), suggesting that the proteolytic cascade of plasminogen activation, a critical step during angiogenesis, can result in an angiogenic switch. It was also found that MMP-9 can cause an angiogenic switch in tumor tissue by releasing VEGF from the matrix (64).

Another example is TGF-β, which exerts both tumor-suppressive and -promoting effects (65). The suppressive effect is due to its ability to upregulate the cyclin kinase inhibitors, causing the inhibition of cell proliferation. However, as the cancer progresses, cyclin kinase inhibitors become refractory to growth inhibition and begin to produce large amounts of TGF-β (65). TGF-β is produced as an inactive pro-form and requires proteolytic conversion by furin or other proteinases, such as MMP-9, to become an active form. MMP-9 can localize at the cell surface by binding to the CD44, a hyaluronan receptor, and then activate TGFβ (66). It was also shown that MT1-MMP and MMP-2 could activate TGF-β1 (67). On the other hand, MMP-2 and MMP-9, and MT1-MMP indirectly modulate TGF-β by cleaving the latent TGFβ-binding protein 1 (LTBP-1), releasing ECM-bound TGF-β (68, 69). Since tumor cells often acquire non-responsiveness to TGF-β, the proteolytic activation of TGF-β by MMPs may exhibit cancer-promoting effects by selectively driving stroma-mediated cancer invasion and metastasis (65). It has also been shown that plasmin can release active TGFβ from the ECM (70).

Although it is not a proteolytic action, the degradation of HS chains in HS proteoglycans (HSPGs), such as syndecans and perlecan, by the hepanase glycolytic enzyme can also release growth factors and is also considered to be an essential modulator of growth factor signaling within the TME.

### 3.4 Processing soluble factors: cytokines and chemokines

Proteinases are known to process cytokines and modify their signals (71). Interleukin 1β (IL-1β) is degraded by MMP-1, MMP-2, MMP-3, and MMP-9, while IL-1α is resistant to these proteolytic enzymes (72). Although both IL-1α and IL-1β bind to the same IL-1 receptor (IL1R) and activate through the same pathway, they are separately encoded proteins with low sequence homology and divergent biological processes, cellular localization, and the mechanisms of activation (73). However, IL-1β was shown to be involved in cancer more than IL-1α. IL1β has two opposite roles in cancer: promoting cancer and suppressing cancer. The tumor-promoting effects of IL-1β are to promote inflammation-driven carcinogenesis and cause tissue damage by upregulating various MMPs. On the other hand, the tumor-suppressing effect is the activation of anticancer immunity (73). Cancer may take advantage of IL-1β degradation to evade immunity.

Another example of proteolytic processing is chemokines. It has been shown that MMP-dependent chemokine proteolysis can affect the biological functions of chemokines in different ways (71). First, the proteolysis inactivates the chemokine. Second, the processing generates antagonistic derivatives, which can still bind to the chemokine receptor but cannot exert chemotaxis. Third, the truncation of chemokine results in a higher chemotactic effect. It has been shown that MMP-1, MMP-2, MMP-3, MMP-9, MMP-13, and MT1-MMP inactivate CXCL12 (stromal cell-derived factor-1) (74). MMP-2 also sheds the plasma membrane-associated chemokine, CX3CL1 (fractalkine), generating a soluble chemokine. However, an additional cleavage at the N terminus of the protein by MMP-2 inactivates the chemokine, converting it into a potent antagonist (75).

MMP-9 also inactivates CXCL chemokines, including CXCL4 and CXCL1 (76). MMP-9 inactivates CXCL5 and CXCL7 as well (76). It was also shown that MMP-8 and MMP-9 inactivate CXCL9 and CXCL10 by processing their C-terminus (77).

Several inactivated chemokines can still bind to their receptors, acting as functional antagonists. MMP-1, MMP-2, MMP-3, MMP-13, and MMP-14 process CCL7 (MCP-3) into an antagonistic form (78, 79). The closely related chemokines CCL2 (MCP-1) and CCL13(MCP-4) can also be cleaved by MMP-1 and MMP-3 and CCL8 (MCP-2) by MMP-3 (79). Thus, MMPs can exhibit anti-inflammatory effects by dampening the action of chemokines.

MMP-9 was shown to process CXCL8 (IL-8), significantly increasing chemotactic activity (76). MMP-8, MMP-13, and MT1-MMP also generate truncated IL-8 species with increased activity (80, 81). MMP-8 also activates CXCL5 (81).

### 3.5 Membrane protein shedding and processing

ECM–cell communication is mediated by cell surface receptors and other cell surface molecules, and the proteolytic cleavage of these membrane proteins termed shedding modifies microenvironment signaling. The major shedding enzymes are MT1-MMP, ADAM17, and ADAM10 in the TME. They are type-I TM proteinases, and, together, these enzymes modify diverse signaling pathways. This section discusses six shedding examples that influence cancer progression, namely, CD44, ICAM-1, DDRs, syndecans, EphA2, and HB-EGF.

#### 3.5.1 CD44

CD44 is a type I TM cell adhesion molecule whose ligand is hyaluronic acid (HA), a glycosaminoglycan (82–84). It has been shown that CD44 can also bind to osteopontin (85), fibronectin, type I collagen (86), type IV collagen (87), and matrix metalloproteinases (MMPs) (88). CD44 is expressed in most cell types in our body, and a shed form of soluble CD44 has been detected in the circulation and other body fluids (83). A single gene encodes CD44, but alternative splicing generates multiple isoforms. CD44 gene contains 20 exons, and the most common form of CD44, referred to as standard or hematopoietic CD44, contains 10 exons (82, 83, 89). This form is the shortest isoform, and other forms have the insertion of alternative exons (V2–V10) at a single site within the membrane-proximal region of the ectodomain (82, 83, 89). Interestingly CD44 with V3 insertion made CD44 to be modified with HS, which may provide additional functionality to the receptor: HB-EGF presentation (90). CD44 consists of N-terminal HA-binding globular domain, followed by a stem with glycosylation and GAG binding sites, a TM domain, and the cytoplasmic tail. The cytoplasmic domain binds to band 3.1 proteins (ERM proteins), linking CD44 to the actin cytoskeleton (83) (**Figure 2A**). It has been reported that CD44 can transition to a high-affinity state upon the stimulation of the cells by soluble factors (91–93). However, the molecular event on CD44 during the activation process is not understood.

CD44 was shed by three TM metalloproteinases, namely, MT1-MMP (94), ADAM10, and ADAM17 (95, 96). The CD44 shedding by each metalloproteinase was shown to promote cell migration (94, 95). MT1-MMP shedding occurs constitutively at the lamellipodia when CD44 and MT1-MMP are coexpressed in the cells (94). It was found that CD44 shedding by MT1-MMP promoted cancer cell migration on the HA-based substratum (94). CD44 interacts with MT1-MMP through its stem region and the hemopexin (Hpx) domain of MT1-MMP, which mediates MT1-MMP localization at the lamellipodia (22). ADAM10- or ADAM17-dependent CD44 shedding was induced by calcium influx or protein kinase C activation, respectively (97, 98). However, when cell migration on the HA-based matrix was measured, the knockdown of ADAM10 or 17 in human lung adenocarcinoma inhibited the migration by 75% in both (98), suggesting that ADAM-dependent CD44 shedding also supports cell migration on the HA matrix. Since CD44 is localized at the lamellipodia, and the suppression of Rac1 by overexpressing Rac1 dominant-negative mutant inhibited the shedding (97), CD44 shedding by these proteinases also occurs at the lamellipodia. It has been reported that adding HA to the cells initiated CD44 shedding (99), suggesting that CD44 shedding may occur at the leading edge where CD44 binds to the HA-containing substratum (**Figure 2**). It was shown that after shedding the ectodomain by a metalloproteinase, the soluble intracellular domain of CD44 was released by presenilin-dependent gamma-secretase (100, 101), and this fragment has a transformation activity (101). Thus, CD44 ectodomain shedding triggers transformation as well. Taking together, the membrane proteinase-dependent shedding is likely the core of CD44-mediated cell migration. As described above, CD44 is cleaved by three membrane-bound metalloproteinases, and all of this shedding promotes cell migration. However, it is unclear which shedding events play a role in different types of cell migration. In human melanoma cells, the constitutive shedding of CD44 was reported to be mediated by ADAM10 but not by MT1-MMP or ADAM17, although all these enzymes are expressed in the cells (102). Further clarifications are required in the future.

### 3.6 Intercellular adhesion molecule–1

Intercellular adhesion molecule (ICAM)–1 is a TM glycoprotein of the immunoglobulin (Ig)−like superfamily. It consists of five extracellular Ig−like domains (D1-D5), a TM domain, and a short cytoplasmic tail. ICAM-1 interacts with the α_L_β_2_ integrin (lymphocyte function-associated antigen 1 or LFA-1), mediating cell–cell interaction. ICAM-1 is expressed in endothelial cells, and α_L_β_2_ integrin in lymphocytes and myeloid cells, and ICAM-1–α_L_β_2_ interaction is crucial for the transendothelial migration of the lymphocytes and myeloid cells (103). Soluble proteolytically shed ICAM-1 (sICAM-1) has been detected in human serum, and it contains all five IgG domains of the D1-D5 (104). Thus, the cleavage for this shedding occurs between D5 and the TM domain. It has been shown that NE cleaves ICAM-1 (105). However, it was revealed that an antibody against D1 inhibited NE-mediated cleavage, indicating that NE is unlikely to cleave between D5 and the TM region. Therefore, NE is unlikely to be responsible for generating sICAM-1. Later, it was found that phorbol 12-myristate 13-acetate-induced ICAM-1 shedding was due to ADAM17 (106). In addition, it was found that MT1-MMP-mediated ICAM-1 shedding plays a crucial role during the transendothelial migration of monocytes (107). It was also reported that oxidative stress–induced ICAM-1 shedding was MT1-MMP dependent (108). Most recently, it has been found that ADAM10-mediated ICAM-1 shedding plays a role in the transendothelial migration of neutrophils (109). Thus, it is possible that ICAM-1 shedding may be involved in the infiltration of lymphocytes and myeloid cells within the TME. ICAM-1–α_L_β_2_ interaction was also shown to play a role in the transendothelial migration of the melanoma (110). It was demonstrated that the coculture of melanoma cells with endothelial cells induced the expression of α_L_β_2_ in melanoma cells, allowing them to interact with ICAM-1 in endothelial cells. Given the role of ICAM-1 shedding during the transendothelial migration of lymphocytes and myeloid cells, it is expected that the shedding also plays a role in the transendothelial migration of melanoma.

#### 3.6.1 Syndecans

Syndecans are type 1 TM HSPGs. The HS chains at the extracellular domains interact with different ligands, including ECM glycoproteins, cytokines, chemokines, and growth factors. There are four syndecans. Syndecan-1 is highly expressed in epithelia, syndecan-2 in endothelia and fibroblasts, syndecan-3 is mainly expressed in neuronal and some musculoskeletal tissue, while syndecan-4 can be found in most tissues. A single cell can express multiple syndecans. Each syndecan is attached by three HS chains, and syndecan-1 and syndecan-3 are attached by additional two chondroitin sulfate chains. The TM domain of all syndecans contains a GXXXG motif that promotes the formation of SDS-resistant dimers (111, 112). This TM domain–mediated dimer was reported to be crucial for the function of syndecan-2 and syndecan-4 (112). Syndecans are known to be shed by many different proteinases (113). Syndecan-1 was shown to be shed by MMP-7 (114), MMP-9 (115), MT1-MMP, MT3-MMP (116), and ADAM17 (117). Syndecan-2 was shown to be shed by MMP-2, MMP-9 (118), MMP-7 (119), and MT1-MMP (120). Syndecan-3 sheddase was shown to be metalloproteinase, but it has not been identified yet (121). Serine proteinase, thrombin, was also shown to cleave syndecan-3 (122). Syndecan-4 is shed by MMP-9 (115), ADAM17 (117), ADAMTS1 (123), plasmin (124), and thrombin (122, 124). Syndecan shedding has two biological effects. First, it decreases syndecan levels on the cell surface. Several growth factors are known to interact with the HS chain of the syndecans, including fibroblasts growth factor (FGF), vascular endothelial growth factor (VEGF), EGF, hepatocyte growth factor (HGF), platelet-derived growth factor (PDGF), and transforming growth factor β1 (TGFβ1). This interaction is essential for growth factor signaling. It has been shown that HS-bound FGF-2 increased the affinity for FGFR by over one magnitude (125). Thus, loss of syndecan by shedding would greatly influence the presentation of growth factors to the receptors. The second effect is that the shed ectodomain of syndecans can act as a soluble factor that exerts biological function. For instance, shed soluble syndecan-1 from fibroblasts can mediate mitogenic responses in human breast cancer cells. This paracrine event is mediated by the HS chain, basic FGF, and stromal-derived factor 1 (126). Another example can be that shed syndecan-2 deposited to the ECM can be a ligand for the protein tyrosine phosphatase receptor CD148 to promote β1 integrin-mediated cell adhesion (127).

#### 3.6.2 Discoidin domain receptors

Discoidin domain receptors (DDRs) are collagen receptor tyrosine kinases, and there are two types, DDR1 and DDR2. Both DDRs have a collagen-binding discoidin domain at the N-terminus of the ectodomain and tyrosine kinase domain at their cytoplasmic domain. DDRs are the only receptor tyrosine kinase whose ligands are collagens. Both DDRs bind to fibrillar collagens, but DDR1 additionally binds to type IV collagen. Under physiological conditions, DDR1 is expressed in epithelial cells, while DDR2 is expressed in mesenchymal cells.

DDR1 and DDR2 bind to the GVMGFO motif (128, 129) found in collagen types I, II, and III, distinct from the β1 integrin-binding site GFOGER (130). Thus, the binding of DDRs and integrins are independent. For DDRs to bind collagens, they must form a homodimer (131). The DDR1 dimer is likely mediated through the leucine zipper in the TM domain (132), while the DDR2 ectodomain spontaneously forms a dimer (131). Thus, ligand binding–induced dimerization, which is found in many receptor tyrosine kinases, does not apply to DDRs. It has been shown that further clustering of dimer DDRs occurs upon collagen binding. Interestingly, inter-DDR dimer phosphorylation was shown to occur between DDR1s and between DDR1 and DDR2 (133).

It was shown that the DDR1 ectodomain is proteolytically shed upon the collagen stimulation of the cells, which can be inhibited by a broad-spectrum metalloproteinase inhibitor (134). Later, the responsible enzyme was identified as ADAM10 (135). Interestingly ADAM10 and DDR1 exist as a stable complex on the cell surface, but the shedding does not occur unless collagen binds to DDR1. Since the interaction of DDRs with collagen cannot be controlled by inside-out signaling like integrins, ectodomain shedding is the only means to dissociate cells from DDR1-mediated collagen adhesion. It was shown that shedding-deficient DDR1 had a much longer half-life of collagen-induced tyrosine phosphorylation (135), suggesting that DDR1 shedding controls the duration of collagen signaling. The DDR1-mediated collagen signal has been shown to increase cell motility (136). When cells migrate on the collagen matrix, adhesion to the matrix is essential, but dissociation from the collagen is equally important, and ADAM10-dependent DDR1 shedding plays a key role. The inhibition of DDR1 shedding by ADAM10 significantly inhibited epithelial cell migration on the collagen matrix (135). Aside from ADAM10, MT1-MMP was also reported to shed a DDR1 ectodomain. It was shown that the coexpression of MT1-MMP with DDR1 in COS7 cells caused a constitutive shedding of DDR1 ectodomain (137). However, this event was not shown in endogenous MT1-MMP and DDR1 (137). Thus, further investigation is necessary to examine the role of MT1-MMP in DDR1 shedding.

DDR2 must also be dissociated from collagen upon transmitting collagen signals, but DDR2 shedding has not been described, and an alternative mechanism has not been identified.

#### 3.6.3 EphA2

Erythropoietin-producing hepatocellular receptor-2 (EphA2) is a member of the Eph receptor kinase family, which is overexpressed frequently in diverse cancer types (138, 139). EphA2 is also overexpressed in various cancer cell lines, such as fibrosarcoma, breast cancer, and ovarian cancer, and high EphA2 levels are correlated with increased malignancy and poor clinical prognosis (140). Furthermore, ectopic expression of EphA2 in a normal mammary epithelial cell line, MCF10A, was enough to confer tumorigenicity in mice (141). However, activation of EphA2 is known to exhibit tumor-suppressive activities (140, 142); thus, the exact mechanism of tumor-promoting activity of EphA2 was unclear. It turns out that the shedding of EphA2 by MT1-MMP was one of the mechanisms promoting cancer. It was shown that both EphA2 and MT1-MMP are upregulated in different invasive breast cancer cells, and silencing the EphA2 or MT1-MMP gene inhibited collagen invasion of the cells. It was found that the proteolytic cleavage of EphA2 by MT1-MMP initiated increased cleaved EphA2 translocation to the intracellular compartment and increased activity of RhoA small GTPase, which, in turn, caused a repulsive effect between cells, and promoted single cancer cell invasion (143). The shedding of EphA2 by MT1-MMP was also found to play other important roles in cancer progression. Without EphA2 cleavage by MT1-MMP, the ligation of ephrin A1 to EphA2 causes a significant inhibition of EGF-ErbB induced phosphor-Erk, phosphor-Akt, anchorage-independent growth, and cell migration (144). Upon EphA2 cleavage by MT1-MMP, this effect was significantly hampered; thus, EphA2 shedding by MT1-MMP converts a tumor-suppressive RTK to an oncoprotein (144).

#### 3.6.4 Heparin-binding epidermal growth factor

Heparin-binding EGF (HB-EGF), a member of the EGF family, transduces extracellular signals *via* ErbB receptors and plays a pivotal role in many physiologic and pathologic processes (145, 146). HB-EGF is also expressed in various human carcinomas such as pancreatic, esophageal, colon, gastric, ovarian, and bladder cancers (147). HB-EGF is synthesized as a type-I TM pro-HB-EGF, and its propeptide is removed by proprotein convertases such as furin during secretion to the cell surface. The ectodomain of HB-EGF comprises a heparin-binding domain containing a core stretch of basic amino acids at its N-terminus, followed by an EGF-like domain and a juxtamembrane domain (145). Membrane-bound HB-EGF can bind to its cognate ErbB receptors expressed in other cells *in trans*, or its ectodomain can be proteolytically cleaved at the juxtamembrane region to become a soluble HB-EGF and ligate ErbB receptors in neighboring cells, transmitting a signal. It has been reported that ADAM10, ADAM12, and ADAM17 shed HB-EGF to generate a soluble HB-EGF. In addition, MMP-2 or -9 (148), MMP-7 (149), and MMP-10 (150) were also reported to shed HB-EGF as well. This shedding event makes HB-EGF bioavailable to cells within the TME.

The heparin-binding domain (HBD) of HB-EGF prevents the EGF-like domain from binding to its cognate ErbB receptors. However, heparin-binding to the HBD renders the EGF-like domain available to ligate ErbB. Thus, the binding to the HSPG is thought necessary for HB-EGF to transmit a signal to the cells. However, it was found that MT1-MMP removes the N-terminal 20 amino acids of HBD by cleaving at A_81_-L_82_, making an HB-EGF heparin-independent growth factor (151). It was shown that this MT1-MMP cleavage plays a significant role in cancer cell growth in a three-dimensional matrix (151).

## 4 Conclusion and future prospective

Within the TME, there are many signaling events in cancer cells and neighboring cells. They can be signals from the ECM, soluble factors, and cell–cell communications. As discussed above, many of these signaling events involve the proteolytic modulation of signaling molecules. Therefore, proteolytic enzymes are considered part of these signaling events, and cancer cells utilize them for their malignant progression. It becomes apparent that several enzymes promote cancer progression by modulating multiple signaling events. Especially, membrane-bound metalloproteinase, MT1-MMP, ADAM10, and ADAM17, are significant players. For instance, MT1-MMP plays a role in ECM degradation for invasion and metastasis, laminin-5 γ2 chain processing to stimulate cell motility and growth, cleaving chemokines to modulate host immunity, shedding CD44 to promote cell migration, shedding ICAM-1 to mediate trans-endothelial migration, shedding EphA2 to enhance EGF signaling in cancer, and cleaving the N-terminus of HB-EGF to convert it to heparin-independent growth factor, promoting cancer cell growth and motility. ADAM10 and ADAM17 play a role in CD44 shedding to promote cell migration, ICAM-1 shedding to mediate the trans-endothelial migration of cells, syndecans’ shedding to modulate heparan-sulfate-mediated signaling events, ADAM10-mediated DDR1 shedding to modulate DDR1-mediated cell adhesion and to control prolonged collagen signaling, and HB-EGF shedding to generate soluble HB-EGF. Thus, the inhibition of each enzyme or all these enzymes would inhibit multiple events in the TME, which significantly impact cancer progression. Therefore, these metalloproteinases can be potentially effective target molecules for cancer therapy. However, the past failures of the clinical trial of metalloproteinase inhibitors have hampered metalloproteinase inhibitor drug development. Further understanding of the regulation of these enzymes during cancer progression may reveal novel means to control the activity of these enzymes without directly inhibiting the enzyme activities so that novel treatments that target cancer invasion, growth, and metastasis can be developed in the future.

## Author contributions

The author confirms being the sole contributor of this work and has approved it for publication.

## Funding

This work was funded by Special fund by the Kennedy Institute of Rheumatology.

## Conflict of interest

The author declares that the research was conducted in the absence of any commercial or financial relationships that could be construed as a potential conflict of interest.

## Publisher’s note

All claims expressed in this article are solely those of the authors and do not necessarily represent those of their affiliated organizations, or those of the publisher, the editors and the reviewers. Any product that may be evaluated in this article, or claim that may be made by its manufacturer, is not guaranteed or endorsed by the publisher.
